# HRQoL in adolescents with idiopathic isolated GHD: rhGH (dis)continuation in mid-puberty

**DOI:** 10.1530/EC-25-0843

**Published:** 2026-02-19

**Authors:** Joeri Vliegenthart, Jan Busschbach, Edmond H H M Rings, Erica L T van den Akker, SEENEZ Growth Hormone Consortium, Danielle C M van der Kaay

**Affiliations:** ^1^ Division of Paediatric Endocrinology, Department of Paediatrics, Erasmus University Medical Centre, Sophia Children’s Hospital, Rotterdam, The Netherlands; ^2^ Section of Medical Psychology and Psychotherapy, Department of Psychiatry, Erasmus MC, Rotterdam, The Netherlands; ^3^ Department of Paediatrics, Erasmus University Medical Centre, Sophia Children’s Hospital, Rotterdam, The Netherlands; ^4^ Division of Pediatric Endocrinology, Department of Pediatrics, Reinier de Graaf Gasthuis, Delft, The Netherlands; ^5^ Division of Pediatric Endocrinology, Department of Pediatrics, Beatrix Children’s Hospital, University Medical Centre Groningen, Groningen, The Netherlands; ^6^ Division of Pediatric Endocrinology, Department of Pediatrics, Willem-Alexander Children’s Hospital, Leiden University Medical Centre, Leiden, The Netherlands; ^7^ Division of Pediatric Endocrinology, Department of Pediatrics, Emma Children’s Hospital, Amsterdam University Medical Center, Amsterdam, The Netherlands; ^8^ Division of Pediatric Endocrinology, Department of Pediatrics, Franciscus Gasthuis & Vlietland, Rotterdam, The Netherlands; ^9^ Dutch Growth Research Foundation, Rotterdam, The Netherlands; ^10^ Division of Pediatric Endocrinology, Department of Pediatrics, Wilhelmina Children’s Hospital, University Medical Center Utrecht, Utrecht, The Netherlands; ^11^ Division of Pediatric Endocrinology, Department of Pediatrics, Jeroen Bosch Hospital, ‘s-Hertogenbosch, The Netherlands; ^12^ Division of Pediatric Endocrinology, Department of Pediatrics, Zuyderland Hospital, Heerlen, The Netherlands; ^13^ Division of Pediatric Endocrinology, Department of Pediatrics, Amalia Children’s Hospital, Radboud University Medical Centre, Nijmegen, The Netherlands; ^14^ Division of Pediatric Endocrinology, Department of Pediatrics, Maastricht University Medical Centre, Maastricht, The Netherlands; ^15^ Division of Pediatric Endocrinology, Department of Pediatrics, Catharina Hospital, Eindhoven, The Netherlands; ^16^ Division of Pediatric Endocrinology, Department of Pediatrics, St. Antonius Hospital, Nieuwegein, The Netherlands; ^17^ Division of Pediatric Endocrinology, Department of Pediatrics, Canisius Wilhelmina Hospital, Nijmegen, The Netherlands

**Keywords:** HRQoL, QoL, quality of life, idiopathic isolated growth hormone deficiency, IIGHD, growth hormone deficiency, GHD, mid-puberty, puberty

## Abstract

**Objective:**

To evaluate health-related quality of life (HRQoL) in adolescents with idiopathic isolated growth hormone deficiency (IIGHD) who tested GH-sufficient, comparing those who discontinued recombinant human growth hormone (rhGH) at mid-puberty with those who continued until near-adult height (NAH).

**Design:**

This multicentre prospective study used a patient-preference design. Previous findings showed that NAH did not differ between groups. Height influences quality of life (QoL), particularly during adolescence when appearance and social comparison affect psychological development. The impact of height and treatment decisions on HRQoL during puberty remains unclear.

**Methods:**

Adolescents with IIGHD who had received rhGH for ≥3 years and tested GH-sufficient in mid-puberty chose to continue or discontinue treatment. HRQoL was assessed at mid-puberty and NAH using QoLISSY (patient and parent reports), supplemented by KIDSCREEN-52, SDQ, and EQ-5D-Y.

**Results:**

Of 127 participants, 44 continued rhGH and 83 discontinued. Questionnaire completion was 58% (*n* = 74) at mid-puberty and 66% (*n* = 84) at NAH. No significant differences in patient-reported QoL were observed between groups at either time point. Parents reported higher QoL in the discontinuation group at mid-puberty. Overall, QoL scores were within normal ranges and positively correlated with height SDS at both time points.

**Conclusions:**

Discontinuing rhGH in adolescents with IIGHD who tested GH-sufficient at mid-puberty does not appear to negatively affect perceived QoL. Parental reports suggest greater well-being in the discontinuation group, possibly reflecting pre-existing satisfaction with height and health. These findings emphasize considering both physical and psychosocial factors in treatment decisions and incorporating patient and parent perspectives during puberty.

## Introduction

Height is considered a relevant factor in shaping an individual’s quality of life (QoL), particularly during childhood and adolescence when physical appearance and social comparison increasingly influence psychological development. Short stature has been associated with reduced health-related quality of life (HRQoL), although the strength and nature of this relationship vary across populations and age groups (1). For example, a large UK population study found that adults with significant height deficits reported notably lower HRQoL, especially those with a height of more than 2 standard deviations (SDs) below the mean (2). In children, however, psychosocial factors, such as peer support and coping strategies, appear to be more influential determinants of QoL and self-esteem than height (3). Furthermore, research has shown that children and their parents often overestimate the child’s height, particularly before the age of 8–9 years, after which children begin to perceive their stature more realistically – suggesting that awareness of short stature and its psychosocial implications develop gradually with cognitive maturation (4). These findings highlight the complex and evolving relationship between height and perceived well-being across developmental stages.

Despite growing recognition of the psychosocial dimensions of short stature, there remains limited evidence on how HRQoL is affected in adolescents with idiopathic isolated growth hormone deficiency (IIGHD) treated with recombinant human growth hormone (rhGH). Current guidelines advise to continue rhGH until near-adult height (NAH). This is especially relevant when adolescents with IIGHD test GH-sufficient in mid-puberty, prompting the clinical question of whether to continue or discontinue rhGH treatment. A recent study by our group showed that in IIGHD adolescents who tested GH-sufficient in mid-puberty, NAH is comparable between those who continued rhGH treatment until NAH and those who stopped treatment at mid-puberty (5).

This study aimed to achieve two primary objectives: first, to conduct a comprehensive assessment of HRQoL among rhGH-treated adolescents diagnosed with IIGHD in childhood that retested GH-sufficient at mid-puberty; second, to investigate potential differences in HRQoL between patients who discontinued rhGH treatment and those who continued rhGH treatment until NAH, with a particular emphasis on analysing QoL trends as participants transition into adulthood.

## Methods

### Subjects/patients

This study (the SEENEZ GH Study) followed a multicentre prospective patient-preference design to assess QoL in adolescents diagnosed with IIGHD undergoing rhGH treatment. QoL was evaluated specifically in mid-pubertal patients who showed normal results on GH provocation testing. The study population included adolescents in mid-puberty who initiated rhGH treatment following a diagnosis of partial IIGHD and received treatment for at least 3 years. Partial IIGHD was defined as a peak GH level between 1.7 and 10 μg/L (5–30 mU/L) in 2 GH stimulation tests. GHD was classified according to the Dutch national protocol developed by the Growth Hormone Advisory Group, which is based on GH peak during stimulation tests and serum IGF-1 and includes 7 categories, with categories 1–4 considered candidates for rhGH treatment (6, 7). Mid-puberty was defined as Tanner stage G3/G4, testicular volume >12 mL, and bone age (BA) between 13 and 16 years in boys and Tanner stage B3/B4 with BA between 11 and 14 years in girls. Subjects were excluded if they had any medical or psychological condition or used medications other than rhGH that could influence growth. Adolescents with a normal GH peak (>20 mU/L = 7 μg/L) upon retesting in mid-puberty were eligible for inclusion. Growth and pubertal development were monitored every 3–4 months in the group continuing rhGH treatment and every 6 months in the group that discontinued treatment. If the decision was made to continue treatment, rhGH was stopped upon reaching NAH (height velocity <2 cm/year).

### Measurements

The QoL of patients and that reported by their parents were assessed using a disease-specific questionnaire developed for individuals with short stature (8). This instrument was administered at two time points: at study inclusion and upon reaching NAH. At inclusion, both patients and their parents received a paper version of the questionnaire by postal mail, accompanied by a return envelope. After the final measurement point, only patients received a digital questionnaire, distributed via Castor EDC (https://www.castoredc.com/). To boost response rates for the final questionnaire, participants received a digital gift card by email after completion.

#### Quality of Life in Short Stature Youth (QoLISSY)

The QoLISSY instrument was used to assess HRQoL in children and adolescents with short stature. Specifically developed for patients with GHD and ISS, the instrument is suitable for children aged 8–18 years and for parents (proxy version) of children aged 4–18 years (8, 9). It has demonstrated satisfactory reliability and validity in previous studies, making it appropriate for use in rhGH treatment contexts regardless of the underlying cause of short stature (9, 10, 11). The questionnaire was translated and validated for use among Dutch children and adolescents (12). Using a 5-point Likert scale and requiring approximately 10–50 min to complete, the child version includes 22 items across 3 core QoL domains (physical, emotional, and social) and 28 additional items on coping, beliefs, and treatment. The parent version includes the same 22 core items, 44 additional items, and 2 parent-specific domains: future and effects on parents (9). Scoring was performed in SPSS using a syntax provided by the authors of the QoLISSY. Total scores per subdomain and overall scores were calculated, reported, and analysed. All scores were transformed from raw scores to 0–100 scores with higher values representing higher QoL. For each age group, the mean and SD were determined using a reference group composed of respondents from five European countries (8, 9). Based on these values, the deviation of each patient’s score from the age- and sex-specific mean was calculated in terms of SD units.

The questionnaire also includes the questions of the KIDSCREEN-52 (13) and Strengths and Difficulties Questionnaire (SDQ) (14, 15), facilitating both self-report and proxy reports (for caregivers).

#### KIDSCREEN-52

The KIDSCREEN is a generic, cross-culturally applicable instrument developed in accordance with international quality standards to assess HRQoL and well-being in children and adolescents aged 8–18 years, with proxy versions available for parents and caregivers (13). The KIDSCREEN-52 provides detailed profile information across ten Rasch-scaled dimensions of HRQoL: physical well-being, psychological well-being, moods and emotions, self-perception, autonomy, parent relations and home life, financial resources, peers and social support, school environment, and social acceptance (bullying) (16). To calculate total scores for each subscale, we used the official SPSS syntax files provided in the KIDSCREEN Manual, which allow for efficient processing of multiple respondents and handling of missing values according to the recommended procedures. Subscale scores were converted to international *T*-values (*M* = 50, SD = 10). These scores allow for comparison with normative data, where values between 40 and 60 are considered within the normal range.

#### SDQ-Dutch

The SDQ is a 25-item screening tool developed by Goodman to assess psychosocial problems, strengths, and their impact on daily functioning in children. The SDQ total difficulties score was calculated by summing the items from 4 subscales, excluding the prosocial behaviour subscale. The total score was not calculated if any subscale score was missing. Recommended cut-off scores for the parent version were available for the total difficulties score and selected subscales (14, 15, 17). A cut-off score of 14 was considered indicative of risk of psychosocial problems up to the age of 14 years, and a cut-off score of 16 was applied from 15 years onward. Scoring was performed using the publicly available SPSS syntax (https://www.sdqinfo.org/c1.html).

#### EQ-5D-Y

The EuroQol EQ-5D-Y instrument is a validated instrument used to assess HRQoL in children and adolescents (18, 19, 20). In this study, the visual analogue scale (VAS) component of the EQ-5D-Y was primarily used to evaluate participants’ self-rated health at inclusion and NAH.

### Statistical analysis

QoL data were compared between groups at the onset of the study and after reaching NAH. Data were analysed using IBM SPSS Statistics (version 29.0.1.0). The Kolmogorov–Smirnov test was used to assess data distribution. Group comparisons were conducted using Student’s *t*-test for normally distributed variables and Mann–Whitney *U*-test for non-normally distributed variables. A two-tailed *P*-value of <0.05 was considered statistically significant. Normally distributed data are presented as mean (SD), whereas non-normally distributed data are reported as median (interquartile range; IQR).

A linear mixed-effects model with random coefficients was used to analyse longitudinal changes in QoL scores and to compare these changes between study groups. The model accounted for individual variability by allowing each patient to have a unique baseline level (intercept) and rate of change over time (slope). The intercept variance represented the degree of variation in initial QoL scores between individuals, while the slope variance reflected differences in the trajectories of QoL over time. The model included time (onset of the study and at NAH), treatment group (rhGH continuation and stop group), and their interaction as fixed effects. The model was estimated using restricted maximum likelihood (REML).

### Missing values

In the paper-based questionnaire at inclusion, missing values were handled by imputing a response in 25 child and 28 proxy questionnaires, provided that at least 80% of the items within a subdomain were completed. If two response options were marked, the most extreme was selected. If no response was given, the mean of the completed items within that subdomain was used. The follow-up questionnaire, which was completed digitally, did not allow for missing responses. For the KIDSCREEN, missing values were handled using the official syntax; for the SDQ-D, no analyses were performed if data were missing.

## Results

In this multicentre patient-preference study, a total of 127 patients participated. Of these, 44 patients chose to continue rhGH treatment (GHcont), while 83 patients chose to stop treatment (GHstop) until NAH. Analysis of baseline characteristics revealed no significant differences in height standard deviation scores (SDS) between the GHcont and GHstop groups (5). The mean NAH-SDS minus target height (TH)-SDS in the GHcont group was −0.171 (0.60), compared to −0.177 (0.62) in the GHstop group (*P* = 0.96).

### QoLISSY at mid-puberty (patients)

**Figure 1:**
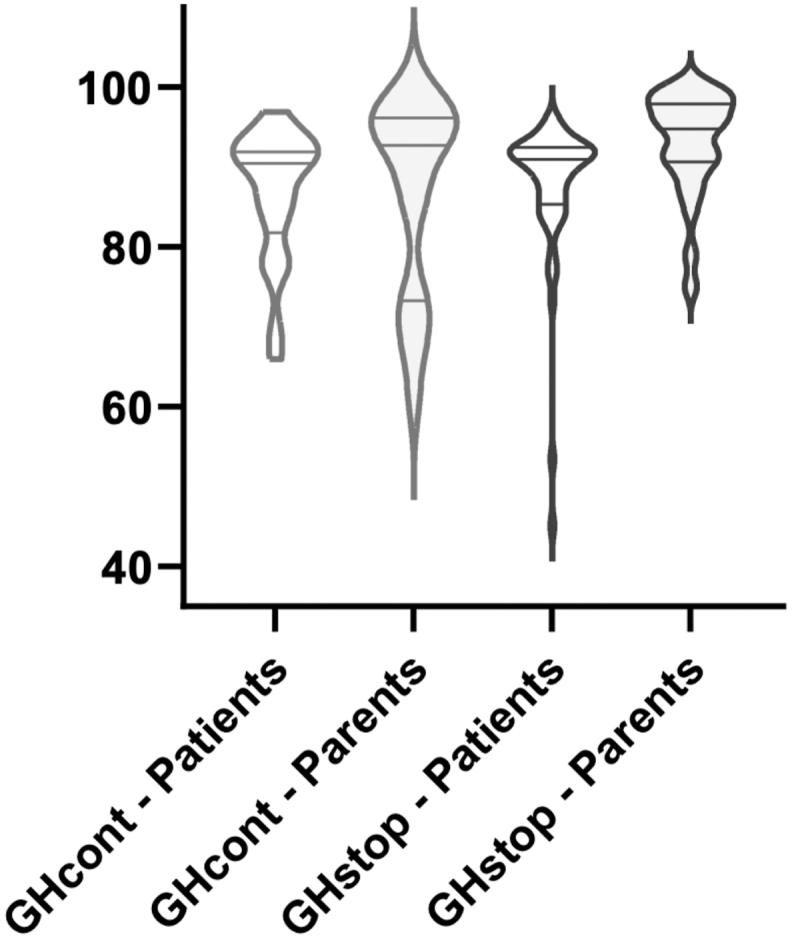
Violin plot showing the distribution of QoLISSY total scores for both GHcont (*n* = 22; 50%) and GHstop (*n* = 52; 63%) groups at mid-puberty, separated by respondents: patients and parents. The plots illustrate the density of the data across the score range, along with median values and overall spread for each category. The horizontal lines within the violin plot indicate the median and quartiles.

Between 2016 and 2022, 74 patients (58% of the total cohort) completed the questionnaires at mid-puberty: 22 respondents (50%) from the GHcont group and 52 respondents (63%) from the GHstop group. No significant differences in patient characteristics (age, height SDS, and TH-SDS) were found between questionnaire responders and non-responders, both at mid-puberty and at NAH. Analysis of patient-reported total QoL scores (Table 1 and Fig. 1) revealed no statistically significant difference between the two groups; the mean (SD) scores were 86.76 (8.28) for the GHcont group and 87.55 (9.20) for the GHstop group (*P* = 0.66). Domain-specific QoL scores did not show significant disparities between the two groups, as shown in Table 2. Moreover, the total QoL scores and domain scores observed in both groups were comparable to age- and sex-adjusted reference values, as shown in Table 2.

**Table 1:** Total QoLISSY-Child scores at mid-puberty and at NAH, presented as median (IQR). ΔQoLISSY-C was calculated as the total QoL score at NAH minus the score at mid-puberty.

	rhGH continuation group		rhGH stop group	
	Median (IQR)	*n* =	Median (IQR)	*n* =
QoLISSY-C at mid-puberty	90.45 (82.99; 91.67)	22	90.97 (85.59; 92.19)	52
QoLISSY-C at NAH	93.81 (81.90; 95.54)	26	93.56 (82.89; 97.92)	58
ΔQoLISSY-C	3.60 (−3.62; 6.18)	16	2.17 (−3.12; 6.60)	42

**Table 2:** Domain scores of the QoLISSY-Child at mid-puberty and comparison of QoLISSY-C total and domain scores to age- and sex-adjusted reference values at mid-puberty. Comparison values represent the mean standard deviation from the reference population. Positive values indicate scores above the reference mean, while negative values indicate scores below the reference mean. *P*-values represent the statistical comparisons between the domain scores.

	rhGH continuation group (*n* = 22)		rhGH stop group (*n* = 52)		*P* =
	Median (IQR)		Median (IQR)		
	Domain score	Comparison with reference	Domain score	Comparison with reference	
Physical	97.92 (87.50–100.00)	0.99 (0.40; 1.09)	100.00 (91.67–100.00)	1.09 (0.63; 1.10)	0.47
Social	96.88 (87.50–100.00)	0.96 (0.49; 1.11)	96.88 (90.63–100.00)	1.02 (0.65; 1.11)	0.73
Emotional	75.00 (71.88–81.25)	0.77 (0.40; 1.05)	78.13 (75.00–78.13)	0.98 (0.69; 1.14)	0.51
Coping	18.75 (15.00–35.00)	−1.54 (−1.86; −0.79)	28.75 (16.25–52.50)	−1.15 (−1.76; −0.31)	0.19
Beliefs	87.50 (68.75–93.75)	0.62 (−0.06; 0.87)	93.75 (81.25–100.00)	0.85 (0.28; 1.08)	0.15
Treatment	51.79 (32.14–64.29)	0.23 (−0.83; 0.88)	61.61 (41.07–69.64)	0.55 (−0.39; 1.02)	0.11
Total QoL score	90.45 (82.99; 91.67)	0.98 (0.39; 1.10)	90.97 (85.59; 92.19)	1.06 (0.73; 1.21)	0.23

### QoLISSY at mid-puberty (parents)

Parental total QoL assessment at inclusion showed a significant difference between the GHcont group (mean (SD) = 85.05 (14.70)) and the GHstop group (mean (SD) = 93.26 (6.36)), with higher scores reported by parents of children who discontinued rhGH treatment (*P* = 0.02). This discrepancy was further evident in the social, emotional, and future domains, where scores were significantly higher in the GHstop group, as shown in Table 3. Parental scores were comparable to age- and sex-adjusted reference values, as shown in Table 3.

**Table 3:** Domain scores of the QoLISSY-Parent at mid-puberty and comparison of QoLISSY-P total and domain scores to age- and sex-adjusted reference values at mid-puberty. Values represent the mean standard deviation from the reference population. Positive values indicate scores above the reference mean, while negative values indicate scores below the reference mean. *P*-values represent the statistical comparisons between the domain scores in the two groups.

	rhGH continuation group (*n* = 22)		rhGH stop group (*n* = 52)		*P* =
	Median (IQR)		Median (IQR)		
	Domain score	Comparison with reference	Domain score	Comparison with reference	
Physical	95.83 (83.33; 100.00)	0.85 (0.21; 1.03)	100.00 (93.75; 100.00)	1.03 (0.74; 1.07)	0.11
Social	92.19 (71.88; 100.00)	0.71 (−0.14; 1.04)	96.88 (90.63; 100.00)	1.04 (0.78; 1.04)	0.02
Emotional	87.50 (65.63; 93.75)	0.81 (−0.13; 1.07)	90.63 (84.38; 96.88)	1.02 (0.75; 1.26)	0.01
Coping	33.75 (12.50; 47.50)	−0.62 (−1.55; 0.04)	27.50 (15.00; 41.25)	−0.90 (−1.50; −0.23)	0.35
Beliefs	78.13 (62.50; 100.00)	0.31 (−0.12; 0.94)	93.75 (78.13; 100.00)	0.93 (0.41; 1.14)	0.06
Treatment	55.36 (50.00; 71.43)	−0.33 (−0.59; 0.37)	66.96 (54.46; 77.68)	0.18 (−0.41; 0.75)	0.10
Future	90.00 (80.00; 100.00)	0.59 (0.18; 0.81)	100.00 (95.00; 100.00)	0.81 (0.80; 1.02)	0.01
Effects on parents	90.91 (79.55; 97.73)	0.89 (0.29; 1.20)	95.45 (90.91; 97.73)	1.10 (0.89; 1.20)	0.09
Total QoL score	92.53 (72.22; 95.83)	0.87 (−0.11; 1.08)	94.97 (90.63; 97.92)	1.06 (0.83; 1.18)	0.02

A positive correlation was found between parents’ total QoL scores and their child’s height SDS at mid-puberty (total group: *r* = 0.605, *P* < 0.001; GHcont: *r* = 0.605, *P* < 0.001; and GHstop: *r* = 0.695, *P* < 0.001).

### QoLISSY at NAH (patients)

Between 2023 and 2024, 84 patients (66% of the total cohort) completed the digital questionnaires at NAH: 26 respondents (59%) from the GHcont group and 58 (70%) from the GHstop group. Mean age at the time of questionnaire completion was 18.7 (1.9) years in the total cohort and did not differ between groups (GHcont: 18.9 (1.8); GHstop: 18.6 (2.0) years; *P* = 0.46). At NAH, total QoL scores were similar between groups, as shown in Table 4, with a mean of 88.1 (10.7) in the GHcont group, compared to 89.8 (11.1) in the GHstop group (*P* = 0.23). Within the domain-specific QoL scores, the beliefs subdomain was significantly higher in the GHstop group (87.50 (81.25; 100.00)) compared to the GHcont group (84.38 (68.75; 93.75); *P* = 0.01), while no differences were observed in the other subdomains. At NAH, all scores were comparable to age- and sex-adjusted reference values, as shown in Table 4.

**Table 4:** Domain scores of the QoLISSY-Child at NAH and comparison of QoLISSY-C total and domain scores to age- and sex-adjusted reference values at NAH. Values represent the mean standard deviation from the reference population. Positive values indicate scores above the reference mean, while negative values indicate scores below the reference mean. *P*-values represent the statistical comparisons between the domain scores in the two groups.

	rhGH continuation group (*n* = 26)		rhGH stop group (*n* = 58)		*P* =
	Median (IQR)		Median (IQR)		
	Domain score	Comparison with reference	Domain score	Comparison with reference	
Physical	95.00 (85.00; 100.00)	0.81 (0.26; 1.09)	95.00 (85.00; 100.00)	0.81 (0.26; 1.09)	0.75
Social	92.86 (82.14; 96.43)	0.76 (0.23; 0.93)	92.86 (82.14; 100.00)	0.79 (0.33; 1.09)	0.26
Emotional	90.63 (78.13; 96.88)	0.69 (0.21; 0.99)	93.75 (84.38; 96.88)	0.84 (0.47; 0.99)	0.24
Coping	32.50 (25.00; 52.50)	−0.90 (−1.22; 0.02)	35.00 (20.00; 52.50)	−0.82 (−1.37; 0.07)	0.72
Beliefs	84.38 (68.75; 93.75)	0.38 (−0.06; 0.62)	87.50 (81.25; 100.00)	0.62 (0.37; 0.87)	0.01
Treatment	69.64 (51.79; 75.00)	0.06 (0.04; 0.07)	58.93 (50.00; 67.86)	0.05 (0.05; 0.06)	0.29
Total QoL score	93.81 (81.90; 95.54)	0.88 (0.28; 0.97)	93.56 (82.89; 97.92)	0.87 (0.35; 1.10)	0.23

### Comparison over time

Comparison of total QoL scores over time showed no significant change in either group (GHcont: *P* = 0.23; GHstop: *P* = 0.32). This finding was confirmed by a random coefficient model based on 158 QoL observations from 100 patients. The average baseline QoL score was 85.65 (SE = 3.86), and scores remained stable over time (*β* = 1.44, SE = 4.86, *P* = 0.77). No significant difference was found between the GH continuation and GH discontinuation groups (difference = 1.19, SE = 2.20, *P* = 0.59), nor in how QoL changed over time between the groups (interaction = −0.09, SE = 2.76, *P* = 0.98). The model used a random intercept variance of 42.79 and a random slope variance for time of 38.31, suggesting substantial individual variability in both baseline QoL and change over time. Although group-level changes were not significant, the model revealed considerable individual variation in both baseline QoL and its trajectory over time.

### Correlation with height SDS

**Figure 2:**
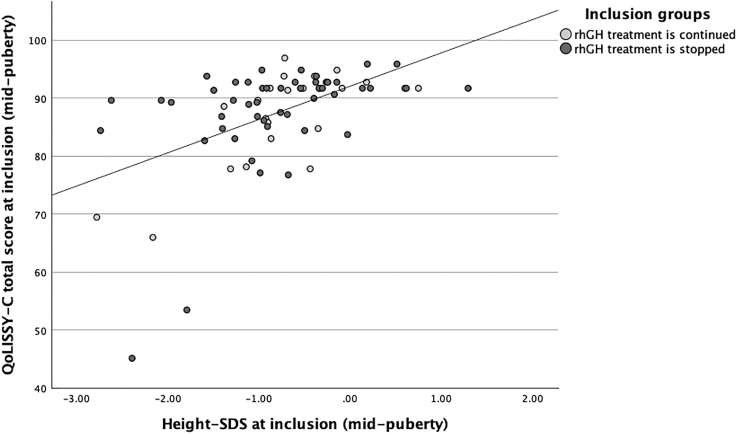
Scatter plot illustrating the relationship between height SDS and QoLISSY-Child (QoLISSY-C) total score at the time of inclusion. Total group: *r*(72) = 0.54, *P* < 0.001; GHcont: *r*(20) = 0.61, *P* = 0.003; and GHstop: *r*(50) = 0.52, *P* < 0.001.

**Figure 3:**
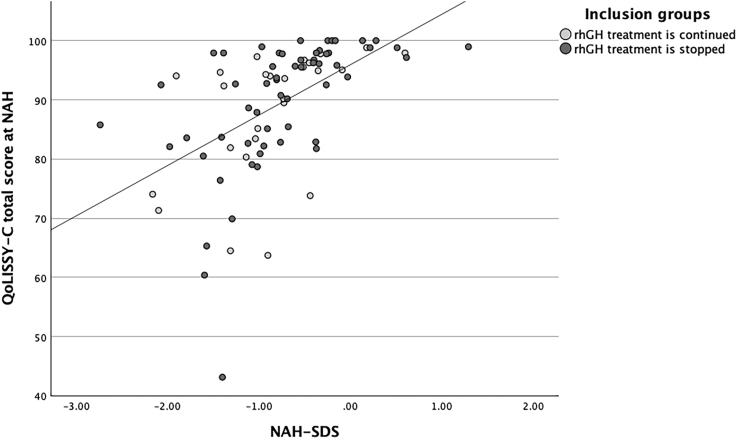
Scatter plot showing the positive correlation between NAH-SDS and total QoLISSY-Child (QoLISSY-C) score at NAH. Total group: *r*(82) = 0.53, *P* < 0.001; GHcont: *r*(24) = 0.51, *P* = 0.008; and GHstop: *r*(56) = 0.54, *P* < 0.001.

In the total group and in both groups individually, positive correlations were found between total QoL score and height SDS (at mid-puberty and NAH), as shown in Figs 2 and 3.

**Figure 4:**
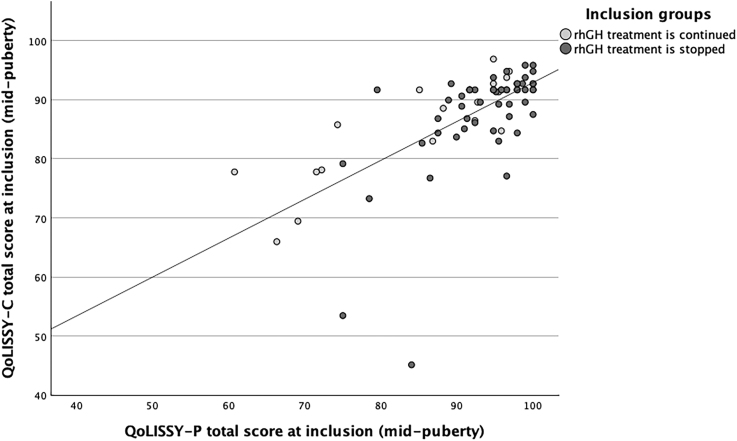
Scatter plot illustrating the positive correlation between total QoLISSY scores reported by children (QoLISSY-C) and their parents (QoLISSY-P) at mid-puberty. Significant correlations were observed in the total sample (*r*(70) = 0.66, *P* < 0.001), the GHcont group (*r* = 0.857, *P* < 0.001), and the GHstop group (*r* = 0.631, *P* < 0.001).

Figure 4 shows that at mid-puberty, a positive correlation was found between the total QoL score reported by children and that reported by parents in the total group (*r* = 0.66, *P* < 0.001), the GHcont group (*r* = 0.857, *P* < 0.001), and the GHstop group (*r* = 0.631, *P* < 0.001).

### EQ-5D-Y

At mid-puberty, mean (SD) EQ-5D-Y VAS scores were 84.47 (11.97) for the GHcont group and 84.29 (10.39) for the GHstop group (*P* = 0.95). At NAH, scores were 85.64 (8.95) for the GHcont group and 80.95 (11.34) for the GHstop group, *P* = 0.06. The change in VAS score (ΔVAS) from inclusion to NAH was −1.76 (12.02) for the GHcont group and −3.23 (15.85) for the GHstop group *P* = 0.74.

### KIDSCREEN-52

The KIDSCREEN-52 self-reported outcomes (Table 5) showed that all subscale scores for both the GHcont and GHstop groups fell within 1 SD of the international mean, indicating overall normative levels of perceived QoL. At inclusion, the GHstop group reported higher scores on the peers subscale than the GHcont group (*P* = 0.047). Proxy-reported outcomes (Table 6), based on parental assessments, also showed scores within the international range. A significant difference was found on the autonomy subscale at inclusion, where parents of children in the GHstop group reported higher autonomy scores compared to those in the GHcont group (*P* = 0.037).

**Table 5:** Self-reported KIDSCREEN-52 subscale scores, converted to international *T*-values (*M* = 50, SD = 10), for the GHcont and GHstop groups, presented at inclusion and NAH. Note: questions related to school and bullying were not included at the NAH time point.

	rhGH continuation group		rhGH stop group	
	Mean (SD)		Mean (SD)	
	Inclusion (*n* = 22)	NAH (*n* = 27)	Inclusion (*n* = 51)	NAH (*n* = 58)
Physical well-being	53.79 (11.44)	52.19 (11.03)	52.45 (10.81)	50.38 (8.36)
Psychological well-being	49.74 (8.13)	49.84 (9.69)	52.35 (8.45)	48.97 (7.02)
Moods and emotions	50.87 (8.80)	46.30 (9.16)	52.59 (10.86)	46.90 (10.86)
Self-perception	53.14 (9.48)	48.51 (7.76)	52.13 (9.27)	47.27 (7.28)
Autonomy	52.20 (8.68)	50.92 (8.08)	52.85 (9.04)	50.64 (6.62)
Parents	52.71 (8.29)	52.58 (7.24)	53.78 (9.19)	50.48 (8.55)
Financial	54.96 (7.54)	55.82 (6.80)	58.37 (6.54	55.20 (7.53)
Peers	48.02 (10.49)*	48.96 (6.76)	53.34 (9.10)*	50.21 (6.09)
School	49.56 (6.46)	-	52.22 (9.63)	-
Bullying	51.81 (9.90)	-	51.42 (9.51)	-

* Significant difference: peers subscale at inclusion between GHcont and GHstop groups (*P* = 0.047).

**Table 6:** Proxy-reported KIDSCREEN-52 subscale scores converted to international *T*-values (*M* = 50, SD = 10) for the GHcont group and the GHstop group, presented at inclusion. Higher scores indicate better perceived QoL as reported by caregivers.

	rhGH continuation group (*n* = 21)	rhGH stop group (*n* = 52)
	Mean (SD)	Mean (SD)
Physical well-being	54.71 (11.04)	49.81 (9.64)
Psychological well-being	51.76 (9.32)	52.43 (9.37)
Moods and emotions	49.48 (12.72)	50.53 (11.20)
Self-perception	51.49 (11.58)	51.91 (9.60)
Autonomy	50.68 (7.44)*	55.01 (8.49)*
Parents	52.34 (10.20)	53.84 (8.51)
Financial	56.73 (9.12)	57.61 (7.05)
Peers	48.84 (11.30)	52.70 (10.92)
School	50.14 (8.43)	52.76 (9.16)
Bullying	49.29 (11.58)	50.65 (10.59)

* Significant difference: Proxy Autonomy subscale at inclusion between the GHcont and GHstop groups (*P* = 0.037).

### SDQ

Mean (SD) SDQ total difficulties score at mid-puberty was 11.10 (5.49) in the GHcont group (*n* = 21) and 8.16 (4.57) in the GHstop group (*n* = 51; *P* = 0.02). At mid-puberty, mean (SD) height SDS was significantly higher (−0.48 (0.75)) in the group scoring below the SDQ cut-off (*n* = 58) vs −1.01 (0.67) in the group scoring above the cut-off (*n* = 14), *P* = 0.018). At NAH, mean (SD) SDQ total difficulties score was 11.04 (4.60) in the GHcont group and 11.04 (5.25) in the GHstop group (*P* = 0.99). Mean (SD) NAH-SDS was −0.80 (0.72) in the group scoring below the SDQ cut-off of 16 (*n* = 71) and −0.76 (0.48) in the group scoring above the cut-off (*n* = 12, *P* = 0.85).

## Discussion

This multicentre prospective study investigated HRQoL in adolescents with IIGHD who tested GH-sufficient in mid-puberty. Our findings revealed no statistically significant differences in patient-reported total QoL scores between GH-sufficient mid-pubertal adolescents who continued rhGH treatment and those who discontinued it, both at mid-puberty and at NAH. Interestingly, an anticipated difference in QoL between the groups could not be demonstrated. It was expected that adolescents who were more satisfied with their height and well-being would be more likely to choose discontinuation.

Notably, parental assessments indicated higher QoL scores in the group that discontinued treatment, particularly in the emotional, social, and future domains. Across both groups, QoL scores were generally comparable to age- and sex-adjusted reference values. A positive correlation was observed between height SDS and QoL in both groups throughout the study.

While previous studies have reported improvements in emotional and social QoL during the first year after starting rhGH treatment (21), we no longer observed this effect in the last phase of treatment. Moreover, we did not find evidence of a decline in QoL following discontinuation of rhGH treatment. Our expectation was that QoL would be higher in the group discontinuing rhGH treatment, speculating that they might already be more satisfied with their height and therefore chose to stop rhGH treatment. We observed no significant differences in average height, age, and TH between the groups at the beginning of the study, indicating good comparability between the groups (5). The absence of significant differences in patient-reported QoL between treatment groups suggests that discontinuation of rhGH in mid-puberty does not adversely affect perceived well-being at group level.

Although the average QoL remained stable across both groups, the model revealed substantial variability between individual patients. This indicates that some patients experienced changes not captured by group-level averages, underscoring the importance of personalized assessment in clinical practice. However, both KIDSCREEN-52 self-reported and proxy-reported outcomes indicated that the QoL scores across all subscales remained within the international reference range, suggesting that, overall, participants in both the GHcont and GHstop groups experienced normal levels of well-being. The consistent correlation between height SDS and QoL underscores the relation of physical stature with self-perception and well-being. Notably, at mid-puberty, patients in the GHcont group had significantly higher SDQ total difficulties scores than those in the GHstop group, indicating a greater risk of psychosocial difficulties among patients who decided to continue rhGH treatment. Furthermore, patients scoring above the SDQ cut-off had a lower height SDS, suggesting a link between shorter stature and greater psychosocial difficulties. By the time participants reached NAH, SDQ scores were comparable between the GHcont and GHstop groups and no significant differences in height SDS were observed based on SDQ scores.

Since the average age at study inclusion (GH-sufficient at mid-pubertal retest) was 14 years, it is uncertain to what extent parents influenced treatment choices during this pubertal phase. Parents in the GHstop group reported significantly higher scores on the KIDSCREEN-52 autonomy subscale, suggesting that children who discontinued rhGH treatment were perceived as more independent. The correlation between child and proxy reports aligns with previous findings, which demonstrated strong agreement between parent and child assessments of both generic and height-specific HRQoL (22). The significant differences in parental perceptions, favouring the GHstop group, may reflect a selection effect, whereby parents who were already more satisfied with their child’s height and overall well-being were more likely to discontinue rhGH treatment. In contrast with our findings, qualitative focus groups conducted during the development of the QoLISSY instrument revealed that children generally rated their QoL lower than their parents (23). Greek children receiving daily rhGH reported good HRQoL, with higher scores in the physical and emotional domains compared to their caregivers, although treatment-related burden was more pronounced in older adolescents (24). Lackner *et al.* found that the majority of parents of children with IGHD experienced mental stress – often related to social pressure, stigmatization, and challenges with rhGH treatment – and emphasized the importance for clinicians to recognize parental burden and consider psychological support when impaired HRQoL is identified (25).

At NAH, a significant difference was observed in the ‘beliefs’ subdomain, with higher scores reported in the GHstop group than in the GHcont group. This subdomain included questions such as ‘I believe that being taller would make me happier’ and ‘I believe that being tall helps in life’; lower scores indicate stronger agreement with these statements, reflecting a greater value placed on height. Interestingly, this difference was not mirrored in the overall or other subdomain scores, suggesting a more nuanced psychological dimension. These findings support former recommendations for multidisciplinary approaches that address both the physical and psychological aspects of short stature (23, 25, 26, 27). Compared to age- and sex-adjusted reference values, most QoLISSY domain scores in both children and parents in our cohort were comparable with or above the normative mean, indicating generally favourable QoL outcomes. This partially aligns with findings from an Italian cohort, which reported significantly elevated scores only in the physical domain, while coping and treatment domains were notably lower among treated GHD patients and their caregivers (28).

A key strength of our study is the inclusion of both patient- and parent-reported QoL assessments, using the widely validated and most commonly applied QoLISSY questionnaire within this field of research, which shows high reliability and sensitivity to changes during rhGH treatment (11, 29). Another strength is the use of a linear mixed-effects model with random coefficients, which has been shown to outperform traditional repeated measures ANOVA and *t*-tests in terms of bias and coverage, particularly when data are missing at random and individual heterogeneity is present (30).

A limitation of this study is the limited response rate, which may affect generalizability. Nonetheless, more than 50% of participants in both groups completed the questionnaire, enabling meaningful comparisons. A recent large response rate study found similar rates (31). Second, due to ethical constraints related to participant age, parental QoL could not be assessed at NAH, which may have restricted the scope of family-cantered insights. Third, cultural and socio-economic factors – known to influence perceptions of QoL – were not explored. Fourth, the potential influence of physicians’ expectations and the way anticipated height outcomes were communicated to participants was not assessed. This may have affected treatment decisions and perceptions of QoL. Future research should aim to apply longitudinal designs that extend into adulthood and continue to integrate both patient and parent perspectives for a more comprehensive evaluation of treatment outcomes.

## Conclusions

This study found no significant differences in patient-reported QoL between IIGHD adolescents who continued and those who discontinued rhGH treatment in mid-puberty when they tested GH-sufficient, suggesting that stopping treatment does not negatively impact perceived well-being. Parental reports indicated higher QoL in the discontinuation group at mid-puberty, possibly reflecting pre-existing satisfaction with height and health. Healthcare providers should strive to open communication and shared decision-making that integrates patient and parental preferences with study findings, while also considering the economic impact of prolonged rhGH treatment on society.

## Declaration of interest

The authors declare that there is no conflict of interest that could be perceived as prejudicing the impartiality of the work reported.

## Funding

This study was funded by ZonMw (project number 837004021).

## Ethics statement

The study was approved by the Medical Research Ethics Committee (MREC) of Amsterdam University Medical Centre (protocol number: NL57916.029.16). The study was conducted in accordance with the Declaration of Helsinki (2013) and the Medical Research Involving Human Subjects Act (WMO). Written informed consent was obtained from both patients and their parents.
